# Smoking cessation rate and factors affecting the success of quitting in a smoking cessation clinic using telephone follow-up

**DOI:** 10.18332/tid/143375

**Published:** 2021-12-20

**Authors:** Jianghua Xie, Rui Zhong, Lei Zhu, Xiaochang Chang, Jianhua Chen, Wei Wang, Lemeng Zhang, Ouying Chen, Xinhua Yu, Yanhui Zou, Yanqun Li

**Affiliations:** 1 Hunan Cancer Hospital, The Affiliated Cancer Hospital of Xiangya School of Medicine, Central South University, Changsha City, China; 2 School of Nursing, Hunan University of Chinese Medicine, Changsha City, China; 3 Department of Geriatrics, The Second Xiangya Hospital of Central South University, Changsha City, China

**Keywords:** smoking cessation, smoking, predictors, smoking cessation clinic, smoking quit rate

## Abstract

**INTRODUCTION:**

China has the largest number of smokers in the world. The great majority of China’s smokers desire to quit smoking (QS); however, the success rate of self-quitting is low. This study investigated the effects of smoking cessation (SC) clinics in a cancer hospital in Hunan province and determined the influencing factors of successful SC.

**METHODS:**

Smokers were recruited to QS by healthcare workers in the SC clinic from February 2015 to February 2019. SC counseling was provided and telephone follow-up was conducted at 1 week, and at 1, 3 and 6 months. Patients who continued SC during the follow-up period were considered to have QS.

**RESULTS:**

Of the 344 patients included in this study, 16.3% QS at one week, 26.5% at one month, 27.6% at three months, and 31.7% at six months. Age ≥60 years, previous SC attempts, immediate quit dates, and telephone follow-up times (3–4 calls) were predictive factors for smokers to SC at six months.

**CONCLUSIONS:**

Age, previous attempts to QS, immediate quit dates and telephone follow-up times were independent predictors of SC success at six months. SC clinics and frequent telephone follow-up improve the success rate of SC, especially in patients who have previously tried to QS or in those who set immediate quit dates.

## INTRODUCTION

Smoking can lead to coronary heart disease, stroke, cancer, chronic obstructive pulmonary disease and other diseases in several organs^1^. The adult smoking rate in China is 26.6% (male: 50.2%, female: 2.1%) and the country has the most smokers in the world (approximately 308 million)^2^. Each year, more than one million people in China die due to smoking, and it is estimated that the number of deaths caused by tobacco will reach 3 million in the year 2050^3^. Therefore, quitting smoking (QS) is an important public health goal for China.

While it is beneficial for smokers to quit smoking at any age, the earlier a person quits, the greater the benefit^4^. The adult smoking cessation (SC) rate in China increased from 2010 (16.9%) to 2018 (20.1%), but it is still at a low level^2,5^. It has been reported that 19.8% of Chinese smokers have tried to QS in the past year; however, most of these attempts failed due to the uncontrollable desire to smoke and nicotine withdrawal symptoms^2^. Behavioral support has been used as part of SC counseling and interventions in SC clinics are low-cost and could double an individual’s likelihood of quitting successfully^6^. A meta-analysis reported that individuals provided with behavioral support for at least six months had a 40–80% increased chance of successfully quitting smoking^7^. In addition, the frequency and intensity of behavioral interventions have a strong dose-response relationship with the success rate of SC^6^, and a telephone follow-up intervention can increase the SC rate^8^.

A number of studies have reported that men, older age, previous attempts to QS, lower Fagerström test for nicotine dependence (FTND) scores, greater motivation to QS, and more consultation sessions are factors related to successful SC^9,10^. Results of a prospective study in six Chinese cities showed that previous SC attempts, willingness to quit immediately and negative opinion of smoking, were factors that predicted making SC attempts, while older age, longer period of SC and willingness to quit immediately were independent predictors of SC success^11^. In Hunan, as elsewhere in inland provinces in China, there has been a lack of data regarding to evaluating the effectiveness of smoking cessation clinic interventions and factors that affect smoking cessation, which is not conducive to the development of smoking cessation clinics and the implementation of effective smoking cessation interventions. In addition, the studies had focused on the factors influencing smokers’ success in QS that needs to be further explored.

Therefore, the purpose of this study was to determine the factors that predict successful SC in an SC clinic in inland China. The roles of telephone follow-up and behavioral support in the process of SC were evaluated, and the level of success was analyzed.

## METHODS

### Study design

This study included smokers treated for SC by medical staff at an SC clinic of a tertiary cancer hospital (Hunan Cancer Hospital) in Hunan Province, China from February 2015 to February 2019. The study followed the CONSORT reporting guideline.

### Participant recruitment

Participants were referred by doctors, recruited in the community or voluntarily visited the SC clinic from February 2015 to February 2019. The inclusion criteria were: age ≥15 years; smoked ≥1 cigarette/day for more than half a year; willing to QS; and able to be contacted by phone. Smokers with life-threatening or serious diseases, cancer, cognitive dysfunction, or incomplete information were excluded from the study.

### Sample size

In this study, the formula of sample size, required by the overall rate to determine the target sample size, was:


$$\text{n}=\frac{u_{a/2}^{2}\pi \;(1-\pi )}{\delta^{2}}$$

According to a previous study^12^ based on behavioral support in Hong Kong, China, the rate of patients who QS for six months was 15.3%, *α* = 0.05, *u_α/2_* = 1.96, δ = 0.05, and the sample size was then calculated to be at least 199. Taking into account a dropout rate of 20%, the estimated sample needed to be at least 239 cases.

### Data collection and evaluation indexes

Baseline data were collected at the patients’ initial visit using the SC clinic questionnaire based on the guidelines for SC clinic practices^13^. The questionnaire items are based on sociodemographic data (including sex, age, education level, and occupation). The patients’ health status was reported by the patients as good, fair, or poor. Each patient’s daily smoking volume, years of smoking, attempts to quit, use of SC products, and start date for SC, were recorded.

Using multiple choice questions, the reasons and motivations to QS were evaluated. For example, patients were asked ‘What is the reason for your decision to QS this time?, with responses: 1) knowing the hazards of smoking, 2) the family members required it, 3) decline in health, 4) knowing somebody sick due to smoking, 5) to avoid trouble in no-smoking places, 6) improve appearance, and 7) other. Patients were encouraged to participate in a CO concentration detector (Micro CO) test at their first assessment; with CO scores of 0–6 ppm indicating non-smoking, 7–10 ppm light smoking, 11–72 ppm heavy smoking, and >72 ppm CO poisoning^13^. The nicotine dependence of each patient was assessed using the FTND score, a six-question survey with a score of 0 indicating no dependence, 1–3 low dependence, 4–6 moderate dependence, and 7–10 high dependence^14^.

### Smoking cessation interventions

Behavioral support was provided via 20 to 30 minutes of face-to-face SC counseling conducted by the medical staff at the first visit. The ‘5As’ and ‘5Rs’ methods proposed by Prochaska and Goldstein in 1991 were used^15^.

After the first visit, four standardized telephone interventions were conducted by medical personnel at 1 week, and at 1, 3 and 6 months after the patient’s visit at the SC clinic^13^. The healthcare providers answered the patients’ questions, provided psychological support and help, and urged patients to remain quit. Each telephone follow-up session lasted 15–20 minutes. A follow-up questionnaire including items regarding smoking status, QS status, withdrawal symptoms, and reasons for failure to QS was completed. Patients with an FTND score of ≥6 were recommended to receive SC medications. Patients who accepted this recommendation were prescribed SC medications at their own expense.

The primary outcome was the rate of continued SC at 1, 3, and 6 months. The duration of maintaining SC was calculated from the beginning of the first consultation to the assessment date, and those who could not attend the SC clinic were assessed by telephone interview, asking how many cigarettes were smoked in the past 1, 3 and 6 months, rather than asking whether they had quit smoking^13,16^. Self-reported abstinence from the beginning of intervention to the evaluation time point was considered SC. Patients were considered lost to follow-up and considered QS failure if they provided a wrong phone number or did not answer the phone call after more than 7 attempts at different time points during the study period^16,17^.

### Statistical analysis

Data entry and analyses were performed using SPSS version 23.0 software (SPSS, Chicago, IL, USA). Demographic data, smoking characteristics, SC and follow-up are described in terms of frequency and percentage. A univariate analysis of SC at 6 months was conducted using the chi-squared test. In the row by column chi-squared test, when the number of cells with theoretical frequency (T) T<1 or 1≤T<5 exceeded 20% of the total number, the Monte Carlo direct calculation probability method was used to calculate the p-value. In the fourfold table chi-squared test, when n≥40 and 1≤T<5, the continuouscorrected chi-squared test was used. Stepwise logistic regression analysis was performed for factors with univariate analysis and statistical significance (α_in_ = 0.05 and α_out_ = 0.10), to screen the factors affecting the success of SC at 6 months. Odds ratios (ORs) and 95% confidence intervals (CIs) were generated using a multivariable analysis. Statistical significance was set at p<0.05.

## RESULTS

### Patient demographics

Of the 351 patients enrolled in the study, 7 were excluded due to incomplete data. The final analysis included 344 patients. Almost all of the patients were male (97.4%, 335/344), and the mean patient age was 45.63±12.34 years (range: 18–80). The most common occupations were enterprise/business/service personnel (39.0%) and farmers (24.4%). Half of the patients (50.6%, 174/344) were in good overall health, 42.7% (147/344) in fair health, and 6.7 % (23/344) in poor health.

### Smoking behaviors

The average smoking duration was 22.87±12.29 years. Over three-quarters of the patients (77.9%) reported smoking for ≥10 years, and 21.5% reported smoking for ≥30 years. The average number of cigarettes smoked per day was 21.93±11.30 (range: 2–71). More than half of the patients (53.5%) reported smoking between 11–20 cigarettes per day, and 81.7% reported smoking >10 cigarettes per day. The average FTND score was 4.32±2.10, and 14.8% of patients were categorized as highly dependent while 49.4% as moderately dependent. Of the 174 patients who underwent CO detection, 63.2% (110/174) were heavy smokers and 15.5% (27/174) light smokers. Overall, 63.4% (218/344) of patients had previously attempted to QS (Table1).

**Table 1:** Clinical characteristics of study participants according to the smoking cessation (CS) status at 6 months follow-up

*Characteristics*	*Total n (%)*	*Unsuccessful quit n (%)*	*Successful quit n (%)*	*χ^2^*	*p*
**Total**, n	344	235	109		
**Gender**				1.432^a^	0.231
Male	335 (97.4)	231 (98.3)	104 (95.4)		
Female	9 (2.6)	4 (1.7)	5 (4.6)		
Age (years), mean ± SD	45.63 ± 12.34			7.202	0.027
<45	148 (43.0)	111 (47.2)	37 (33.9)		
45–59	144 (41.9)	95 (40.4)	49 (45.0)		
≥60	52 (15.1)	29 (12.4)	23 (21.1)		
**Education level**				0.973	0.808
Primary school or less	29 (8.4)	22 (9.4)	7 (6.4)		
Middle school	109 (31.7)	75 (31.9)	34 (31.2)		
High school	141 (41.0)	95 (40.4)	46 (42.2)		
College or above	65 (18.9)	43 (18.3)	22 (20.2)		
**Occupation**				2.554	0.635
Government/institution staff	58 (16.9)	38 (16.2)	20 (18.3)		
Enterprise/business/service personnel	134 (39.0)	94 (40.0)	40 (36.7)		
Farmers	84 (24.4)	60 (25.5)	24 (22.1)		
Retired/unemployed	33 (9.6)	19 (8.1)	14 (12.8)		
Other	35 (10.1)	24 (10.2)	11 (10.1)		
**Health assessment**				5.522	0.063
Well	174 (50.6)	125 (53.2)	49 (45.0)		
Fair	147 (42.7)	99 (42.1)	48 (44.0)		
Poor	23 (6.7)	11 (4.7)	12 (11.0)		
**Daily cigarette consumption,** mean ± SD	21.93 ± 11.30			2.931	0.231
≤10	63 (18.3)	38 (16.2)	25 (22.9)		
11–20	184 (53.5)	126 (53.6)	58 (53.2)		
≥21	97 (28.2)	71 (30.2)	26 (23.9)		
**Smoking duration** (years), mean ± SD	22.87 ± 12.29			1.246	0.742
≤10	76 (22.1)	52 (22.1)	24 (22.0)		
11–20	98 (28.5)	71 (30.2)	27 (24.8)		
21–30	96 (27.9)	63 (26.8)	33 (30.3)		
≥31	74 (21.5)	49 (20.9)	25 (22.9)		
**Fagerström score,** mean ± SD	4.32 ± 2.10			3.165	0.205
≤3	123 (35.8)	77 (32.8)	46 (42.2)		
4–6	170 (49.4)	120 (51.1)	50 (45.9)		
≥7	51 (14.8)	38 (16.1)	13 (11.9)		
**CO test results** (ppm)				0.126	0.939
0–6	37 (21.3)	26 (21.5)	11 (20.8)		
7–10	27 (15.5)	18 (14.9)	9 (17.0)		
11–72	110 (63.2)	77 (63.6)	33 (62.2)		
**Previous SC attempts**				6.905	0.009
Yes	218 (63.4)	138 (58.7)	80 (73.4)		
No	126 (36.6)	97 (41.3)	29 (26.6)		
**Date of SC**				15.112	0.004
Undecided	65 (18.9)	50 (21.3)	15 (13.8)		
Immediately	97 (28.2)	56 (23.8)	41 (37.6)		
Within 7 days	23 (6.7)	13 (5.5)	10 (9.2)		
Within 30 days	103 (29.9)	69 (29.4)	34 (31.2)		
Over 30 days	56 (16.3)	47 (20.0)	9 (8.2)		
**SC reasons and motivation** (multiple selection)					
Knowing the hazards of smoking	251 (73.0)	178 (75.7)	73 (67.0)	2.905	0.088
Decline in health	86 (25.0)	53 (22.6)	33 (30.3)	2.368	0.124
Family asks to quit	81 (23.5)	54 (23.0)	27 (24.8)	0.133	0.716
Knowing somebody sick due to smoking	53 (15.4)	41 (17.4)	12 (11.0)	2.368	0.124
Avoid smoking in non-smoking areas	19 (5.5)	14 (6.0)	5 (4.6)	0.268	0.605
Improve appearance	18 (5.2)	14 (6.0)	4 (3.7)	0.392^a^	0.531
Other	22 (6.4)	15 (6.4)	7 (6.4)	0.000	0.989
**SC aids**				-	0.358*
BS	331 (96.3)	228 (97.0)	103 (94.5)		
BS and medication	7 (2.0)	3 (1.3)	4 (3.7)		
BS and EC	6 (1.7)	4 (1.7)	2 (1.8)		
**Follow-ups**				5.392^a^	0.020
≤2	24 (7.0)	22 (9.4)	2 (1.8)		
3–4	320 (93.0)	213 (90.6)	107 (98.2)		

a Continuous correction value.

* Monte Carlo. BS: behavioral support. EC: electronic cigarettes.

### Reasons and motivations to QS

**Figure 1:**
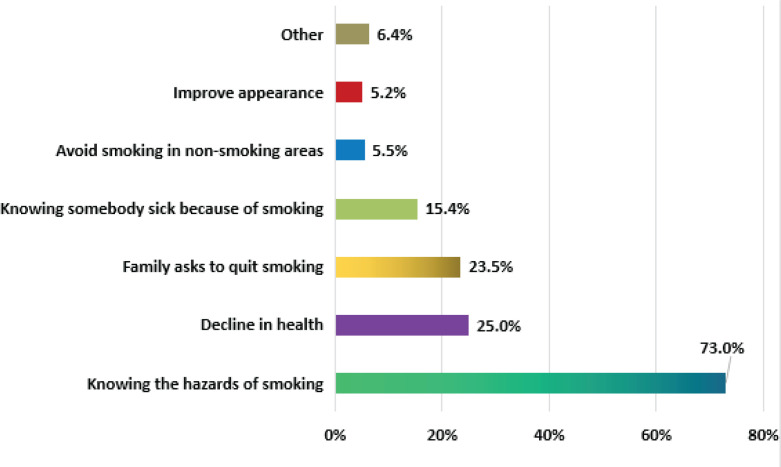
The reasons given as a percentage of the 344 smokers who chose to quit

Awareness of the hazards of smoking (73.0%; 251/344) and a gradual decline in health caused by smoking (25.0%; 86/344) were the two most common reasons for quitting smoking provided by the patients. Other reasons given were: the family members requested it, knowing somebody sick due to smoking, avoiding smoking in no-smoking places, and improving appearance (Figure 1).

**Figure 2:**
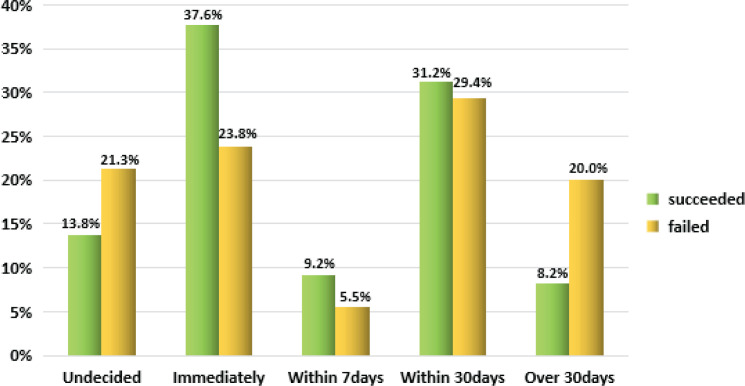
The percentage distribution of the expected start time of smoking cessation between the successful and unsuccessful smoking cessation groups

Ninety-seven patients (28.2%) chose to QS immediately, while 23 (6.7%) chose to QS within 7 days, 103 (29.9%) set a SC date within 30 days, 56 (16.3%) set a date after 30 days, and 65 (18.9%) did not set a SC date (Figure 2).

### Follow-up and SC rate

**Figure 3:**
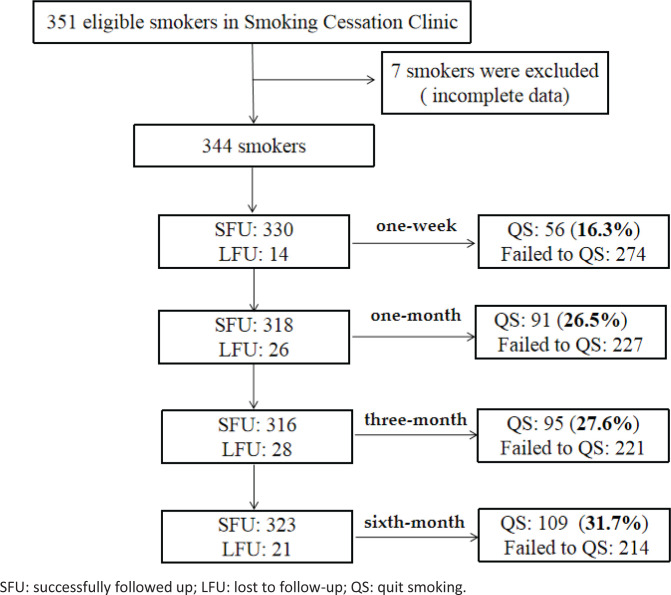
Flowchart for follow-up and successful smoking cessation at each time point

Participants were followed up 1–4 times. Of the 344 patients that were followed up, 16.3% QS at 1 week, 26.5% at 1 month, 27.6% at 3 months, and 31.7% at 6 months (Figure 3).

Behavioral support was provided to 96.3% (331/344) of patients, and 93.0% (320/344) of patients received 3–4 follow-up visits by 6 months. In the SC success group, 98.2% (107/109) of participants received 3–4 follow-ups. For the SC failure group, this was 90.6% (213/235). Only 3.7% (13/344) of the total patients received medication or e-cigarettes in addition to behavioral support (Table 1).

### Reasons for failure to QS

The main reasons for the patient’s failure to quit smoking were: withdrawal symptoms (50.9%), influence of smoking friends (25.0%), work pressure (11.4%), negative emotions such as tension and anxiety (7.0%), and no confidence to quit (3.1%).

### Factors impacting SC success at six months

No significant differences in sex, education level, occupation, health assessment, daily smoking amount, smoking duration, FTND score, CO level, reasons and motivations for quitting smoking, or SC method, were found between the success group and the failure group. Patient age (p=0.027), previous attempts to QS (p=0.009), quit date (p=0.004), and the number of follow-up calls (p=0.020) were significantly different between the two groups (Table 1).

### Independent predictors of SC success at six months

The patient’s SC status was used as the dependent variable, while age, previous SC attempts, quit date, and number of follow-ups were used as independent variables, and stepwise logistic regression analysis was performed.

The results showed that age, previous SC attempts, quit date, and number of follow-up calls were independent predictors of SC at six months. Patients aged ≥60 years were more likely to QS than those aged <45 years (OR=2.72; 95% CI: 1.343–5.512, p=0.005). Patients with previous SC attempts were more likely to quit than those who had not previously tried to quit (OR=2.02; 95% CI: 1.189–3.414, p=0.009). Patients who chose to quit immediately were more likely to be successful than those who were not ready to quit at their first visit (OR=2.26; 95% CI: 1.094–4.668, p=0.028). Patients who had three or four follow-up visits were more likely to QS than those with two or fewer follow-up visits (OR=5.35; 95% CI: 1.207–23.697 , p=0.027) (Table 2).

**Table 2:** Duality logistic regression analysis of potential predictors for smoking cessation (SC) at 6 months follow-up

*Variable*	*β*	*SE*	*Wald χ^2^*	*p*	*OR*	*95 % CI*
**Age** (years)						
<45 (Ref.)					1	
45–59	0.494	0.271	3.319	0.069	1.639	0.963–2.791
≥60	1.001	0.360	7.724	0.005	2.721	1.343–5.512
**Previous SC attempts**						
No (Ref.)					1	
Yes	0.701	0.269	6.786	0.009	2.015	1.189–3.414
**Date of SC**						
Undecided (Ref.)					1	
Immediately	0.815	0.370	4.851	0.028	2.260	1.094–4.668
Within 7 days	0.859	0.539	2.539	0.111	2.360	0.821–6.788
Within 30 days	0.370	0.374	0.982	0.322	1.448	0.696–3.011
After 30 days	-0.521	0.482	1.168	0.280	0.594	0.231–1.528
**Follow-ups**						
≤2 (Ref.)					1	
3–4	1.667	0.760	4.873	0.027	5.348	1.207–23.697

## DISCUSSION

This study confirmed the effectiveness of SC intervention in an SC clinic in mainland China, and the important role of telephone follow-up in increasing the SC rate. In the analysis of predictors of SC, age ≥60 years, previous SC attempts, immediate quit date, and telephone follow-up, were associated with SC success. The number of telephone follow-up calls is a significant factor affecting the success of SC; the more telephone follow-up sessions, the higher the possibility of QS.

### The relationship between age and successful SC

Previously studies have suggested that age is an independent predictor of SC success^18^. In this study, patients aged ≥60 years were more likely to QS than patients aged <45 years. A cross-sectional secondary analysis of data from the India Global Tobacco Survey^19^ revealed that younger patients had a lower number of SC attempts and a lower SC rate compared to patients aged >50 years. Meanwhile, older patients who have smoked for a longer period of time are at higher risk for smoking-related diseases and are more likely to encounter health problems that motivate patients to QS^20^.

### Motivation to QS

Health concerns are the most common reason provided by patients who attempt to QS. An awareness of the health hazards of smoking and gradual decline in QS motivated patients in this study. Previous studies have suggested that patients with worsened health due to smoking have more motivation to quit^21^. The motivation to QS is an important factor in predicting SC attempts, success and continuity, and that a higher level of motivation to QS can predict short-term and long-term success^22^. Providing health hazard information to smokers could improve patients’ awareness of the health hazards of tobacco, increase their willingness to QS, and effectively reduces smoking rates^23^. Therefore, medical personnel should focus on educating patients regarding the harmful effects of tobacco and improving the patients’ willingness and motivation to QS. The inclusion of more warning pictures and health information on tobacco should also be considered.

### Previous attempts to QS

Patients who had previously attempted to QS were more likely to succeed than those trying for the first time. Previous attempts to QS were identified an independent predictor in this study, which is consistent with the results of previous studies in China and Europe^11,24^. Chaiton et al.^25^ reported that an average of 30 attempts to QS is associated with the success of QS at one year or longer. The amount of time spent trying to QS is also closely related to the success of QS, as several studies have reported that trying to QS for more than six months is associated with an increased probability of successful quitting compared to attempting to QS for less than six months^11,26^.

### Quitting date

The decision to QS immediately has been identified as an independent predictor of successful SC. In our study, patients who chose to quit immediately were twice as successful as those who were not prepared to quit. A previous study reported that patients in the preparation and action periods of quitting are more likely to QS than those who are considering quitting^27^. Another study reported that patients who made personal decisions to QS immediately were more likely to quit than those who set a quit date in the future^28^. Together, these results highlight the importance of encouraging patients to QS as soon as possible and to help them QS shortly after visiting SC clinics.

### Frequency and intensity of counseling

The number of face-to-face counseling sessions and telephone counseling sessions are important indicators of SC success^29^. In this study, patients who received three or four follow-up sessions were five times more likely to QS than those who received two or fewer sessions. The number of visits to an SC clinic is an important factor in determining SC success. A clinical trial conducted in Turkey reported that patients who were counseled three times or more had a higher rate of quitting smoking^30^. Intensive telephone counseling by SC professionals significantly improves the success rate of SC. A comparative effectiveness test conducted by Sherman et al.^31^ found that patients who received seven telephone counseling sessions had a higher quitting rate than patients who received one or two counseling sessions. However, another study reported that in participants who were given five telephone follow-up visits, the SC rate was highest soon after the first counseling session and gradually decreased over time at 1, 3, 6, and 12 months^32^. Therefore, frequent, intense follow-up sessions are recommended to help patients QS. The duration and frequency of follow-up sessions can be adjusted based on an individual patient’s SC progress and its duration.

### Effects of SC clinics

The SC rates improved gradually from the first week to the sixth month, and were higher than the reported SC rates of the first SC clinic in Guangzhou^27^. In the two recent SC programs in Thailand, the participant cessation rate at 6 months ranged from 12.6– 25.62%^33,34^. Healthcare workers at a primary care tuberculosis clinic in Tswane, South Africa, who gave participants a brief SC intervention, had a sustained SC rate of 21.5% at six months^35^. Therefore, the effects SC clinics, even in developing countries areas, is reaffirmed.

### Limitations

This study is not without limitations. Successful SC was self-reported via follow-up telephone calls without further confirmation using biochemical indicators. This may have resulted in inaccurate data. A small number of patients selected SC medications interventions, resulting in a lower use rate of SC medications, which may potentially have affected the estimates of efficacy.

## CONCLUSIONS

The rate of patients quitting smoking increased gradually from one week to six months, and the more frequent follow-up telephone calls, the more likely patients were to quit. Older age, previous attempts to QS, and immediate quit dates were predictive factors for successful SC. Therefore, medical staff at outpatient SC clinics should attempt to increase patient motivation, encourage patients to set immediate quit dates, and conduct frequent follow-up visits to improve the SC rate.

## Data Availability

The data supporting this research are available from the authors on reasonable request.

## References

[1] National Center for Chronic Disease Prevention and Health Promotion (US) Office on Smoking and Health (2014). The Health Consequences of Smoking—50 Years of Progress: A Report of the Surgeon General.

[2] Chinese Center for Disease Control and Prevention (2019). Global Adult Tobacco Survey in China in 2018. In Chinese.

[3] Chen Z, Peto R, Zhou M (2015). Contrasting male and female trends in tobacco-attributed mortality in China: evidence from successive nationwide prospective cohort studies. Lancet.

[4] Pirie K, Peto R, Reeves GK, Green J, Beral V (2013). The 21st century hazards of smoking and benefits of stopping: a prospective study of one million women in the UK. Lancet.

[5] Chinese Association on Tobacco Control Global Adult Tobacco Survey in China in 2010. In Chinese.

[6] 2008 PHS Guideline Update Panel, Liaisons, and Staff (2008). Treating tobacco use and dependence: 2008 update U.S. Public Health Service Clinical Practice Guideline executive summary. Respir Care.

[7] Lancaster T, Stead LF (2017). Individual behavioural counselling for smoking cessation. Cochrane Database Syst Rev.

[8] Wu L, He Y, Jiang B (2016). Effectiveness of additional follow-up telephone counseling in a smoking cessation clinic in Beijing and predictors of quitting among Chinese male smokers. BMC Public Health.

[9] Hagimoto A, Nakamura M, Morita T, Masui S, Oshima A (2010). Smoking cessation patterns and predictors of quitting smoking among the Japanese general population: a 1-year follow-up study. Addiction.

[10] Hymowitz N, Cummings KM, Hyland A, Lynn WR, Pechacek TF, Hartwell TD (1997). Predictors of smoking cessation in a cohort of adult smokers followed for five years. Tob Control.

[11] Li L, Feng G, Jiang Y, Yong HH, Borland R, Fong GT (2011). Prospective predictors of quitting behaviours among adult smokers in six cities in China: findings from the International Tobacco Control (ITC) China Survey. Addiction.

[12] Abdullah AS, Mak YW, Loke AY, Lam TH (2005). Smoking cessation intervention in parents of young children: a randomised controlled trial. Addiction.

[13] (2008). Control Office of Chinese Center for Disease Control and Prevention. Guidelines for Smoking Cessation Clinics. In Chinese.

[14] World Health Organization (1998). Guidelines for controlling and monitoring the tobacco epidemic.

[15] Prochaska JO, Goldstein MG (1991). Process of smoking cessation. Implications for clinicians. Clin Chest Med.

[16] Hu Y, Xie J, Chang X (2021). Characteristics and Predictors of Abstinence Among Smokers of a Smoking Cessation Clinic in Hunan China. Front Public Health.

[17] Hale JW, Lewis C, Nazir N (2020). One-Time Education Sessions to Help American Indian Smokeless Tobacco Users Quit. J Community Health.

[18] Monsó E, Campbell J, Tonnesen P, Gustavsson G, Morera J (2001). Sociodemographic predictors of success in smoking intervention. Tob Control.

[19] Srivastava S, Malhotra S, Harries AD, Lal P, Arora M (2013). Correlates of tobacco quit attempts and cessation in the adult population of India: secondary analysis of the Global Adult Tobacco Survey, 2009-2010. BMC Public Health.

[20] Ho KS, Choi BW, Chan HC, Ching KW (2016). Evaluation of biological, psychosocial, and interventional predictors for success of a smoking cessation programme in Hong Kong. Hong Kong Med J.

[21] Tucker JS, Shadel WG, Golinelli D, Seelam R, Siconolfi D (2020). Motivation to quit cigarettes and alternative tobacco products: prevalence and correlates among youth experiencing homelessness. J Behav Med.

[22] Jardin BF, Carpenter MJ (2012). Predictors of quit attempts and abstinence among smokers not currently interested in quitting. Nicotine Tob Res.

[23] Evans AT, Peters E, Shoben AB (2017). Cigarette Graphic Warning Labels Are Not Created Equal: They Can Increase or Decrease Smokers' Quit Intentions Relative to Text-Only Warnings. Nicotine Tob Res.

[24] Girvalaki C, Filippidis FT, Kyriakos CN (2020). Perceptions, Predictors of and Motivation for Quitting among Smokers from Six European Countries from 2016 to 2018: Findings from EUREST-PLUS ITC Europe Surveys. Int J Environ Res Public Health.

[25] Chaiton M, Diemert L, Cohen JE (2016). Estimating the number of quit attempts it takes to quit smoking successfully in a longitudinal cohort of smokers. BMJ Open.

[26] Li L, Borland R, Yong HH (2010). Predictors of smoking cessation among adult smokers in Malaysia and Thailand: findings from the International Tobacco Control Southeast Asia Survey. Nicotine Tob Res.

[27] Zhu WH, Yang L, Jiang CQ (2010). Characteristics of smokers and predictors of quitting in a smoking cessation clinic in Guangzhou, China. J Public Health (Oxf).

[28] West R, Sohal T (2006). "Catastrophic" pathways to smoking cessation: findings from national survey. BMJ.

[29] Zhu N, Lin S, Cao C, Xu N, Yu X, Chen X (2020). Nomogram to predict successful smoking cessation in a Chinese outpatient population. Tob Induc Dis.

[30] Esmer B, Sengezer T, Aksu F, Özkara A, Aksu K (2019). Clinical, sociodemographic and tobacco-use factors associated with smoking cessation rates at three years follow-up, Ankara, Turkey. Tob Prev Cessat.

[31] Sherman SE, Link AR, Rogers ES (2016). Smoking-Cessation Interventions for Urban Hospital Patients: A Randomized Comparative Effectiveness Trial. Am J Prev Med.

[32] Fidan F, Pala E, Ünlü M, Sezer M, Kara Z (2005). Factors Affecting Smoking Cessation and Success Rates of The Treatment Methods Used. Sigara Bırakmayı Etkileyen Faktörler ve Uygulanan Tedavilerin Başarı Oranları. The Medical Journal of Kocatepe.

[33] Aung MN, Yuasa M, Moolphate S (2019). Effectiveness of a new multi-component smoking cessation service package for patients with hypertension and diabetes in northern Thailand: a randomized controlled trial (ESCAPE study). Subst Abuse Treat Prev Policy.

[34] Pirompanich P, Jirapramukpitak T, Saiphoklang N (2017). Assessment of a New Smoking Cessation Program at Thammasat University Hospital, Pathum Thani, Thailand. Southeast Asian J Trop Med Public Health.

[35] Louwagie GM, Okuyemi KS, Ayo-Yusuf OA (2014). Efficacy of brief motivational interviewing on smoking cessation at tuberculosis clinics in Tshwane, South Africa: a randomized controlled trial. Addiction.

